# An Entropic Model for the Assessment of Streamwise Velocity Dip in Wide Open Channels

**DOI:** 10.3390/e20010069

**Published:** 2018-01-17

**Authors:** Domenica Mirauda, Marilena Pannone, Annamaria De Vincenzo

**Affiliations:** School of Engineering, Basilicata University, Viale dell’Ateneo Lucano 10, 85100 Potenza, Italy

**Keywords:** dip phenomenon, entropy, river flow, field velocity measurements

## Abstract

The three-dimensional structure of river flow and the presence of secondary currents, mainly near walls, often cause the maximum cross-sectional velocity to occur below the free surface, which is known as the “dip” phenomenon. The present study proposes a theoretical model derived from the entropy theory to predict the velocity dip position along with the corresponding velocity value. Field data, collected at three ungauged sections located along the Alzette river in the Grand Duchy of Luxembourg and at three gauged sections located along three large rivers in Basilicata (southern Italy), were used to test its validity. The results show that the model is in good agreement with the experimental measurements and, when compared with other models documented in the literature, yields the least percentage error.

## 1. Introduction

The velocity dip position is a ubiquitous feature of open channel flows, and describes the phenomenon by which maximum streamwise velocity is detected below the free surface. Many research studies have measured time-mean velocity profiles in steady and uniform flows, and it was commonly accepted that the streamwise mean velocity deviates from the classical log law near the free surface if the aspect ratio of the channel (i.e., the ratio *Ar* of free surface width *B* to average flow depth *H*) is less than a certain value. This phenomenon was first observed about a century ago by [1,2], and further experimental studies showed that it was induced by the presence of secondary cross-sectional flow structures. 

The study by [3] identified two categories of steady secondary currents in fluid flows. The secondary currents of the first kind, or skew-induced streamwise vorticity, which originate from the mean flow and are driven by the curvature effect, e.g., [4], can easily be explained and understood. Secondary currents of the second kind, whose origin is more complex, are observed in straight and non-circular channels as a consequence of turbulence related to the formation of bed ridges, e.g., [5]. Such corner-induced secondary currents consist of upward flow moving into the apex of the corner, and backward flow moving away from the corner along the channel boundaries. The corner vortices are typically damped within a short distance from the sidewalls, as experimentally shown by [6]. Cross-sectional flow structures can also be generated without corners due to lateral (or span-wise) variations in bed topography and roughness, which can trigger the formation of secondary currents independently of the sidewall effect [7,8]. The inception of secondary circulation (either corner or bed-induced) is still being investigated and debated. In case of steady one-directional flows, the vorticity cross-sectional equation, which governs the behaviour of the secondary currents, is [9]:

(1) $$v\frac{\partial \Omega }{\partial y}+w\frac{\partial \Omega }{\partial z}=\frac{\partial^{2}}{\partial y\partial z}\left(<{v^{\prime }}^{2}>-<{w^{\prime }}^{2}>\right)+\left(\frac{\partial^{2}}{\partial z^{2}}-\frac{\partial^{2}}{\partial y^{2}}\right)<v^{\prime }w^{\prime }>+\nu \nabla^{2}\Omega$$

where Ω is the cross-sectional vorticity:

(2) $$\Omega =\frac{\partial w}{\partial y}-\frac{\partial u}{\partial z}$$

*u*, *v,* and *w* are the time-mean velocities along the streamwise (*x*), vertical (*y*), and span-wise (*z*) directions, respectively; *v’* and *w’* indicate vertical and span-wise velocity fluctuations; and *υ* is the kinematic viscosity. The term on the left-hand side of Equation (2) is the rate of vorticity change due to convection of fluid where the vorticity is not uniform; the third term on the right-hand side represents the rate of vorticity change due to molecular diffusion; the first and second term on the right-hand side have no counterpart in the equation of momentum, which gives vorticity variations a peculiar character. Einstein and Li [10] ascribed the origin of corner-related secondary currents in straight channel flows to the first term on the right-hand side of Equation (1) (i.e., to the unbalanced normal Reynolds stresses in the cross-sectional plane), the other terms being negligible.

The mechanism of generation of the secondary currents proposed by [10] is not universally accepted, and was questioned by many researchers, including [11,12,13,14]. Nezu and Nakagawa [7] remarked that “the mechanisms for initiation and maintenance of cellular secondary currents in wide channel flows are not yet well understood.” The role of the channel boundary in the generation of the secondary currents has been widely analyzed. Rodriguez and Garcia [15] detected multicellular structures in a very wide channel with smooth sidewalls and rough bed. The generation of these multicellular structures is attributed to the large roughness gradient between the smooth glass wall and gravel bed, according to the experimental observations by [16]. The study by [17] also implied that the secondary currents may be an effect of bed roughness heterogeneity.

The theoretical investigation by [18] focused on the mechanism of initiation and development of second kind secondary currents, based on the analysis of Reynolds equations in the boundary region. They found that the secondary currents originate from the lateral variation in streamwise velocity (∂*u*/∂*z* ≠ 0), which is directly related to the transverse Reynolds shear stress −*ρ*<*u’w’*> (with *u’* indicating streamwise velocity fluctuation). Rather than the main flow region, the source of the secondary currents is thus represented by the near-bed region. Any small disturbance in the near-bed flow region may trigger secondary currents, which subsequently result in turbulence anisotropy in the main flow region. Hence, the boundary is not only the primary source of turbulence, but also the source of any secondary flow structure. In other words, without external forcing, all of the sustainable eddies come from the boundary as a consequence of velocity variation: ∂*u*/∂*x* ≠ 0 yields large-scale eddies; ∂*u*/∂*y* ≠ 0 yields small-sized eddies or turbulence; and ∂*u*/∂*z* ≠ 0 yields the secondary currents. The more intense secondary flow is observed at the location where the near-bed velocity *u* changes abruptly (e.g., from floodplain to main channel). Conversely, secondary currents are not observable where *u* remains laterally unchanged.

The present study provides an analytical solution for ensemble mean and variance of streamwise velocity dip position, due to cross-sectional vorticity and secondary currents, based on the maximisation of the related information entropy, e.g., [19]. A recent morpho-hydrodynamic application of Shannon’s entropy can be found in [20]. Based on a set of laboratory experiments [21], the paper focusses on the investigation of the cross-sectional velocity entropy in the very peculiar case of gravel braided rivers, where the bifurcation of the flow and the presence of large emerging bars are mapped by the periodic-like maximisation of that variable along the channel axis. Specifically, the reduction in free flow section is associated with local minima of velocity entropy and reduction in secondary circulation. Other applications of the entropic approach are documented in the field of environmental fluid mechanics, e.g., [22,23,24]. Kitanidis [22] and Pannone and Kitanidis [23] respectively introduce and apply a dilution index expressed in terms of concentration distribution entropy for the investigation of groundwater pollutant dynamics. Pannone and De Vincenzo [24] extend the approach by [22] to solute dispersion in backwater river flows, where the stream deceleration is associated with a gradual decline in solute concentration entropy and dilution. Finally, the principle of maximum entropy has also been used as an effective approach for stochastic generation of stream flows and hydrological variables [25].

A number of empirical, analytical, and numerical models were elaborated to predict the velocity dip position in open channels. Wang et al. [26] derived a regression law between dip position along the central cross-sectional axis of narrow channels and aspect ratio. Yang et al. [27] proposed an empirical model for smooth uniform open channels, and detected the velocity dip position near the sidewall region, even for high values of the aspect ratio. A few years later, Absi [28] changed this model into a simple dip-modified log wake law, which made it applicable also to rough wall flows. In 2008, Bonakdari et al. [29] proposed a sigmoid model, later validated by the same authors [30,31], which comes from the ratio of two different functions of *Ar*, and is applicable to both smooth wide and narrow open channels. The model did not satisfy the asymptotic boundary conditions specified by [32], who suggested that the velocity dip position along the central cross-sectional axis of open channels ranges from 0.5*h*_max_ (where *h*_max_ is the flow depth in which the peak is detected) for *Ar* → 0 to *h*_max_ for *Ar* → ∞. In 2013, Guo [33] proposed an empirical model for smooth rectangular open channels, showing that dip position varies exponentially from flow depth to half of the flow depth as the aspect ratio decreases from infinity (wide channels) to zero (pipe flow). In the same year, Pu [34] presented an empirical model for wide and narrow open channels with rough or smooth beds that satisfied both the asymptotic boundary conditions.

Among the numerical studies, Wang and Cheng [8] calculated the velocity dip position by applying the zero turbulent shear stress condition. Sarma et al. [35] derived a velocity binary law, which combines an inner region log law and an outer region parabolic law. They found that the junction point of log and parabolic law is located at 0.5*h*_max_ when the maximum velocity is detected on the free surface. Conversely, if the maximum velocity occurs below the free surface, the junction point height decreases from 0.5*h*_max_ to 0 with the dimensionless dip position. The method fails if no satisfactory tangential parabola is obtained. Guo and Julien [36] described a method to determine the dip position by fitting a parabola to the velocity data near the free surface.

The study by [37], which was proposed as an improvement of [29], investigates the related assumptions and analytical properties by a sensitivity analysis, and provides a more complete evaluation of model accuracy based on the comparison with experimental data sets. Additionally, the parameters of the model and the associated dip positions are discussed in terms of physical meaning and practical implications. The main limitation of the method resides in the need to calibrate a shape parameter as a function of the specific channel characteristics and flow conditions.

Most of the above-mentioned models are affected by other important limitations: they are often only representative of either smooth or rough bed flow; they can only be applicable to the central cross-sectional axis of the channel; and finally, being empirical laws, they are valid for a restricted range of flow conditions.

The present study proposes a general theoretical model to compute the velocity dip position in open channels, based on a probabilistic approach originating from the maximisation of the related information entropy. Other models based on the entropy theory were previously proposed in the literature to detect the maximum velocity. As an example, Chiu and Tung [38] derived an empirical logarithmic relation between the dimensionless dip position and a representative entropy parameter, based on a large number of laboratory and field data collected under various flow and channel conditions that included both steady and unsteady flows. Recently, Kundu [39] obtained a relationship between velocity dip position and aspect ratio that is not related to a specific velocity distribution. However, although it is derived from a theoretical approach and, thus, it is in principle valid under different flow conditions, the model can only be applied to the central cross-sectional axis of open channels for relatively small aspect ratios (0.1552 ÷ 11.8951). Additionally, it contains empirical coefficients that need to be modified ad hoc according to the experimental data under consideration. 

The method proposed by the present study proves to be applicable under different flow and bed conditions, and provides the maximum cross-sectional dip as well as the corresponding velocity value.

## 2. Formulation

In the case of continuous statistical distributions, Shannon’s entropy [19] is mathematically defined as:

(3) H(X)=−∫p(X)ln[p(X)]dX

where *p*(*X*) indicates the probability density function of the variable under consideration. 

The conditional maximisation of open-channel cross-sectional velocity entropy along the local flow depth *h*, carried out by the method of the calculus of variations, e.g., [40], is thus expressed by:

(4) $$H\left(u\right)=-\int_{0}^{u_{\max}}p\left(u\right)\ln\left[p\left(u\right)\right]du$$

subject to the following constraints:

(5) $$\int_{0}^{u_{\max}}p\left(u\right)du=1$$

and

(6) $$\int_{0}^{u_{\max}}up\left(u\right)du=\bar{u}$$

In the above equations, *u* = *u*(*y*) is the depth variable velocity, *u*_max_ is the maximum of the distribution, and $\bar{u}$ indicates local depth average. The result is:

(7) $$p\left(u\right)=\exp\left(\lambda_{1}-1\right)\exp\left(\lambda_{2}u\right)={\left(h\frac{du}{dy}\right)}^{-1}$$

and:

(8) $$H\left(u\right)=-\lambda_{1}+2-u_{\max}\exp\left(\lambda_{1}-1+\lambda_{2}u_{\max}\right)$$

where:

(9) $$\exp\left(\lambda_{1}-1\right)=\frac{\lambda_{2}}{\exp\left(\lambda_{2}u_{\max}\right)-1}$$

(10) $$\bar{u}=\frac{u_{\max}\exp\left(\lambda_{2}u_{\max}\right)}{\exp\left(\lambda_{2}u_{\max}\right)-1}-\frac{1}{\lambda_{2}}$$

and *λ_i_* indicate the Lagrangian multipliers.

Note that Equation (7) implies a cumulative distribution function *F*(*u_y_*) = *P*(*u* ≤ *u_y_*) = *y*/*h*, and thus, *u*_max_ = *u*(*h*).

Based on these results, and after suitable algebraic transformations and generalisations, Chiu [41] derived the following cross-sectional velocity distribution:

(11) $$u\left(y,z\right)=\frac{u_{\max}}{M}\ln\left\{1+\left[\exp\left(M\right)-1\right]\frac{\eta \left(\xi \right)-\eta_{0}}{\eta_{\max}-\eta_{0}}\right\}$$

In Equation (11), *η* is a special function defined so that, in the more realistic case of two dimensional (2-D) cross-sectional distribution, *u* increases monotonically with it, and *η*_0_ and *η*_max_ indicate the values of *η* where *u* = 0 and *u* = *u*_max_, respectively. The so-called “entropic parameter”, *M*, is related to the Lagrangian multipliers by the following expressions:

(12) $$\lambda_{1}=1+\ln\left\{\frac{\left[M\exp\left(M\right)-\exp\left(M\right)+1\right]}{U{\left[\exp\left(M\right)-1\right]}^{2}}\right\}$$

and:

(13) $$\lambda_{2}=\frac{M}{u_{\max}}$$

where *U* (section-averaged velocity) replaces $\bar{u}$. The entropy-related velocity dip position statistics (ensemble mean and variance) are here derived by analogy as it follows.

Let us consider:

(14) $$Y_{d}=\frac{y_{\max}}{h_{\max}}$$

where *y*_max_ is the vertical coordinate of the cross-sectional peak velocity, and *h*_max_ is the maximum cross-sectional depth. Since the minimum observed *y*_max_ is *h*_max_/2, e.g., [32], *Y_d_* cross-sectional entropy can be expressed as:

(15) $$H\left(Y_{d}\right)=-\int_{0.5}^{1}p\left(Y_{d}\right)\log\left[p\left(Y_{d}\right)\right]dY_{d}$$

Switching to the normalised shifted variable:

(16) $$Y_{d}^{\prime }=\left(Y_{d}-0.5\right)/0.5$$

one obtains:

(17) $$H\left(Y_{d}\right)=0.5H\left(Y_{d}^{\prime }\right)=0.5\int_{0}^{1}p\left(Y_{d}^{\prime }\right)\log\left[p\left(Y_{d}^{\prime }\right)\right]dY_{d}^{\prime }$$

Note that $H\left(Y_{d}^{\prime }\right)$ (the entropy of a normalised variable that oscillates between 0 and 1) is formally identical to the normalised velocity (*u*/*u*_max_) entropy:

(18) $$\begin{array}{l} \int_{0}^{1}p\left(Y_{d}^{\prime }\right)\log\left[p\left(Y_{d}^{\prime }\right)\right]dY_{d}^{\prime }=\int_{0}^{1}p\left(\frac{u}{u_{\max}}\right)\log\left[p\left(\frac{u}{u_{\max}}\right)\right]d\left(\frac{u}{u_{\max}}\right)\\ =1+\ln\left\{\frac{\exp\left(M\right)-1}{M}\right\}-\frac{M\exp\left(M\right)}{\exp\left(M\right)-1} \end{array}$$

As a consequence, the probability density functions that maximise them must be formally the same. Therefore, from (8)–(13), with $u_{\max}$ replaced by 1:

(19) $$p\left(Y_{d}^{\prime }\right)=\exp\left(a_{1}\right)\exp\left(a_{2}Y_{d}^{\prime }\right)$$

where:

(20) $$a_{2}=\frac{M}{Y_{d\max}^{\prime }}=M$$

and:

(21) $$\exp\left(a_{1}\right)=\frac{M}{\exp\left(M\right)-1}$$

Once the probability density function of $Y_{d}^{\prime }$ has been determined, one can straightforwardly calculate the corresponding ensemble mean:

(22) $$<Y_{d}^{\prime }>=\int_{0}^{1}Y_{d}^{\prime }p\left(Y_{d}^{\prime }\right)dY_{d}^{\prime }=\frac{M}{\exp\left(M\right)-1}\left[\exp\left(M\right)\left(\frac{1}{M}-\frac{1}{M^{2}}\right)+\frac{1}{M^{2}}\right]$$

and variance:

(23) $$\begin{array}{l} \sigma_{Y_{d}^{\prime }}^{2}=\int_{0}^{1}{\left(Y_{d}^{\prime }-<Y_{d}^{\prime }>\right)}^{2}p\left(Y_{d}^{\prime }\right)dY_{d}^{\prime }=\\ \frac{M\exp\left(M<Y_{d}^{\prime }>\right)}{\exp\left(M\right)-1}\{\exp\left[M\left(1-<Y_{d}^{\prime }>\right)\right][\frac{{\left(1-<Y_{d}^{\prime }>\right)}^{2}}{M}-\\ \frac{2\left(1-<Y_{d}^{\prime }>\right)}{M^{2}}+\frac{2}{M^{3}}]-\exp\left(-M<Y_{d}^{\prime }>\right)\left(\frac{<Y_{d}^{\prime }{>}^{2}}{M}+\frac{2<Y_{d}^{\prime }>}{M^{2}}+\frac{2}{M^{3}}\right)\} \end{array}$$

Anti-transforming the variable:

(24) $$<Y_{d}>=0.5<Y_{d}^{\prime }>+0.5$$

and:

(25) $$\sigma_{Y_{d}}^{2}=0.25\sigma_{Y_{d}^{\prime }}^{2}$$

In terms of field application, the input data needed by our model are:

- Flow rate *Q* (usually provided by upstream hydrometric stations);
- Cross-section shape and area *A* (the use of a simple water-level dipstick is sufficient to reconstruct it);
- Bed grain size;
- A very reduced number of velocity measurements at pre-fixed points within the cross-section.

The method simultaneously provides, with no need for calibration or the use of empirical relationships, the exact value of the maximum velocity, and its exact position within the cross-section. This is how it should work:

Start from a theoretically-based value of *u*_max_ [42]:

(26) $$u_{\max}=\frac{u_{*}}{k}\ln\left(\frac{R_{H}}{2D_{50}}\right)+8.5u_{*}$$

where $u_{*}=\sqrt{gR_{H}i_{f}}$ indicates shear velocity, $R_{H}$ is the hydraulic radius, *g* is the acceleration due to gravity, $D_{50}$ is the representative grain size, *i_f_* is the average bed slope, and *k* = 0.41 is the von Kàrmàn constant. Equation (26) is justified through the hydraulic radius representing the depth of the equivalent 2-D flow, for which the (monotonic) Prandtl logarithmic profile is a robust estimator. Compute the section-averaged velocity *U* from continuity *U* = *Q*/*A*. Compute the tentative parameter *M* from Equations (10) and (13), with *U* replacing $\bar{u}$. Compute the tentative expected dip position from Equations (22) and (24). Measure the velocity along the deepest vertical in correspondence of $<Y_{d}>$. If the value considerably differs from $u_{\max}$, repeat the procedure until convergence is achieved. The iterative process acts by minimizing the objective function, with the optimal value of maximum velocity that is achieved according to:

(27) $$\frac{\left|u_{\mathrm{maxobs}}-u_{\mathrm{maxest}}\right|}{u_{\mathrm{maxest}}}\leq \varepsilon$$

where *ε* is the maximum admitted relative error (here assumed equal to 0.001), and subscripts “*obs*” and “*est*” stand for observed and estimated, respectively. In our tested case studies, convergence was achieved by a number of iterations always smaller than four. Note that a different iterative procedure was recently proposed by [43] based on Chiu’s entropic velocity profile [41], an empirical closure for the dip and surface velocity survey. The target of the iterative method by [43] is not the exact evaluation of the velocity dip position, but rather the channel flow rate, for a given distribution of dip in the flow area, and accurate experimental observation of velocities. In particular, the procedure starts from a value of the velocity dip position obtained by empirical formulas documented in the literature, and ends when the observed cross-sectional ratio of mean to maximum velocity is nearly equal to the estimated counterpart.

## 3. Field Experiments

The model proposed by the present study was tested against field data that were collected, according to ISO 748 [44], at three ungauged sections located along the Alzette river in the Grand Duchy of Luxembourg, and at three gauged sections located along three large rivers in Basilicata, southern Italy.

**Figure 1:**
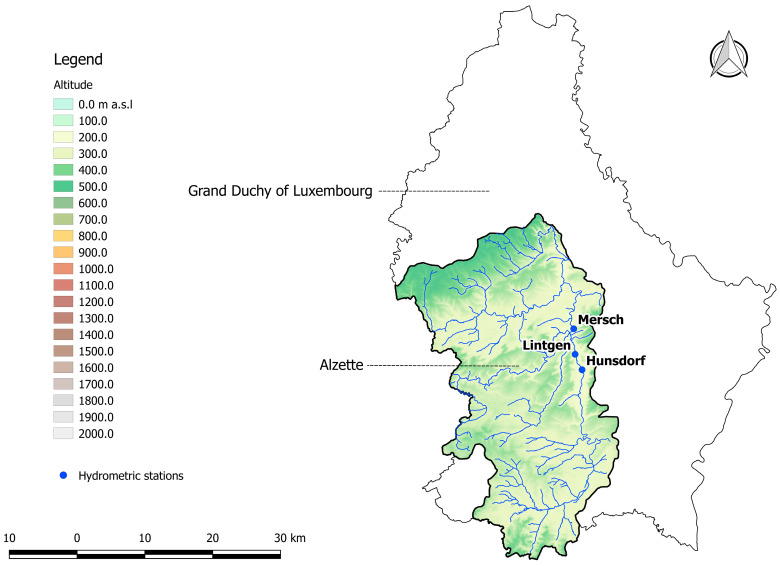
Ungauged sections along the Alzette river.

The Alzette river originates in France at about 4 km from the frontier with Luxembourg. It is the main tributary of the Sûre flowing into the Moselle, which is in turn a tributary of the Rhine. The Alzette river is characterised by a seasonal variation in flow: high levels in winter and low levels in summer, with minimal levels in September. The three measurement sections were located in the northern part of the river, that is, in the valley where the slope exhibited values of about 1% (Figure 1).

**Figure 2:**
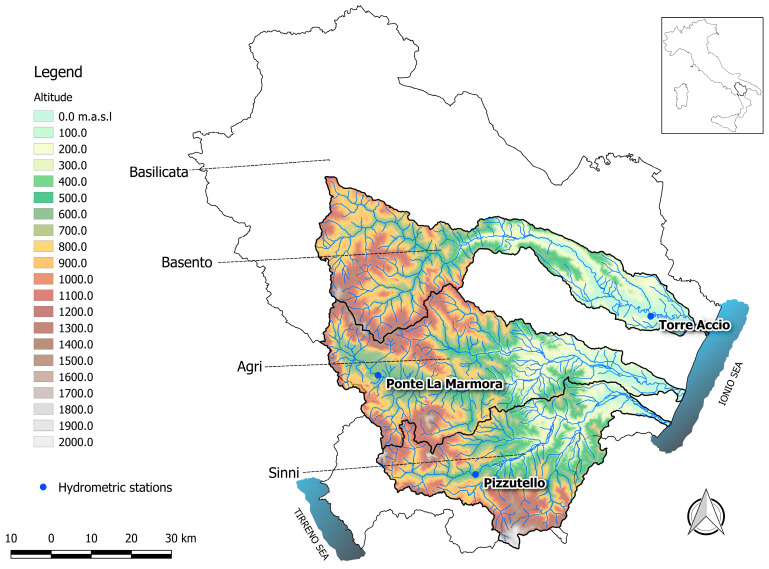
Gauged stations along the Basento, Agri, and Sinni rivers.

The first section, named Hunsdorf, was located along a meandered branch of the river characterised by a gravel bed and pier banks. The second section, named Lintgen, was characterised by pier banks and fine sand/silt bed. The third section, named Mersch, was located along a regularised reach with banks covered by reinforced concrete and fine sand/silt bed. The three sections located in Basilicata were, instead, hydrometric stations. Figure 2 shows the basins of the Basento, Agri, and Sinni rivers along with the position of the corresponding gauged sections.

The first equipped site, named Torre Accio, was located in the valley meandering part of the Basento river. The banks and the bed were made of fine sand and silt. The second station, named Ponte La Marmora, was located in the upper part of the Agri river. Here, the reach under investigation was straight, with a gravel bed and banks covered by reinforced concrete. The third section, named Pizzutello, was located in the middle-upper part of Sinni, where the river exhibited a plan pattern tending to alternating bars. The banks and the bed were made of gravel. 

The velocity measurements at Torre Accio, Ponte La Marmora, and Pizzutello were performed using a Seba Hydrometrie type F1 current meter, with a propeller diameter of 0.080 m and a 0.300 m pitch. The measurements at Hunsdorf and Lintgen were carried out using an OTT type C31 current meter, with a propeller diameter of 0.125 m and a 0.250 m pitch. In conditions of non-accessibility of the cross-section or in case of flood, an automated mobile trolley system of the current meter lowering stabilised by a heavy lead weight (25 or 50 kg) was used, allowing the measurements to be carried out from the bridge. Finally, the RIO Grande ADCP, with a velocity accuracy equal to ±0.25% of the water + boat velocity (±0.25 cm/s), a velocity resolution of 0.1 cm/s and a velocity range ±3 m/s–±20 m/s, was used at the Mersch cross-section. The distance between measurements along the vertical was 0.1 m, while the transverse distance between verticals was set according to the mobile equipment velocity. Table 1 shows the observed ranges of mean depth and flow rate at the six measurement sites.

## 4. Results and Discussion

Table 2 lists the ranges of section-averaged velocity, maximum velocity, entropic parameter, and related entropy for the six investigated measurement sections. 

As one can see, the entropic parameter *M* at each measurement site is characterised by relatively reduced variability as compared with that characterising the flow rate. This is in agreement with recent experimental studies that, investigating a number of gauged sections located along natural channels, e.g., [45,46], and laboratory flumes in steady flow conditions [47,48,49], have found that *M* is almost constant for variable flow rate when the cross-section keeps the same geometry and roughness, while it changes with river axis curvature [45,46] and in conditions of large-scale roughness [47,48,49]. Specifically, Chiu and Said [45] showed that, for a variety of flow rates and depths, a channel cross-section seems to have the propensity to establish and maintain an equilibrium state that corresponds to a defined value of the entropic parameter. Xia [46], after investigating some equipped sites along the Mississippi river, found that the entropic parameter was almost constant along straight reaches (*M* = 2.429–2.442), while it changed slightly with the radius of curvature in meandering reaches (*M* = 4.926–5.017). Greco et al. [47] and Greco and Mirauda [48,49], analyzing data from laboratory steady flows, pointed out that the entropic parameter is considerably affected by riverbed conditions only in cases of large-scale roughness. In the investigated cross-sections, riverbed conditions affected the value of the entropic parameter for low values of flow rate only, thus justifying its variability for the observed *Q* ranges. Thus, once the rigorous determination of *Y_d_* and *u*_max_ was performed for the average *Q*, it could be considered approximately representative of most hydrological events within the investigated flow-rate range.

**Figure 3:**
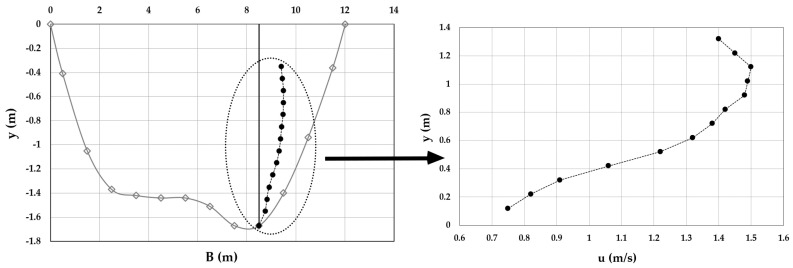
Velocity profile at the Mersch cross-section for *Q* = 16.2 m^3^/s.

**Figure 4:**
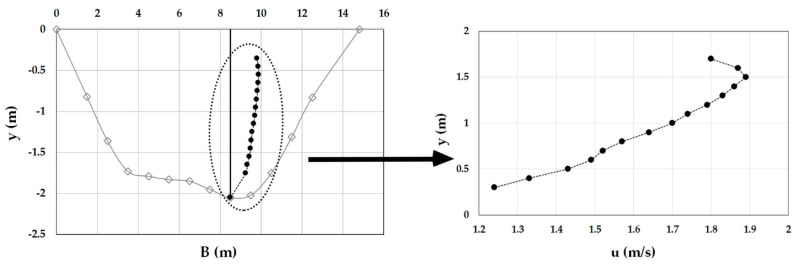
Velocity profile at the Mersch cross-section for *Q* = 26.0 m^3^/s.

**Figure 5:**
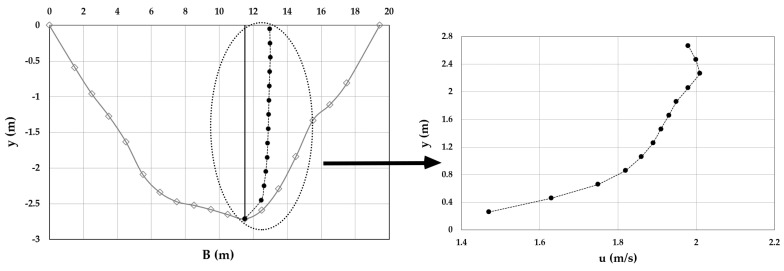
Velocity profile at the Mersch cross-section for *Q* = 44.3 m^3^/s.

**Figure 6:**
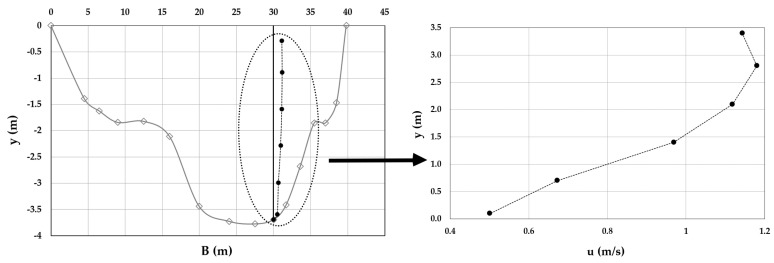
Velocity profile at the Torre Accio cross-section for *Q* = 75.5 m^3^/s.

**Figure 7:**
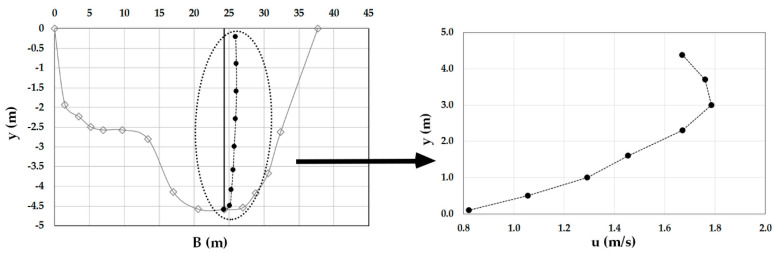
Velocity profile at the Torre Accio cross-section for *Q* = 121.3 m^3^/s.

**Figure 8:**
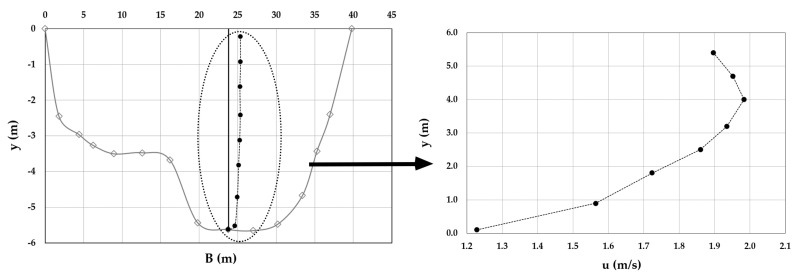
Velocity profile at the Torre Accio cross-section for *Q* = 197.9 m^3^/s.

As an example, Figure 3, Figure 4, Figure 5, Figure 6, Figure 7 and Figure 8 show the velocity profiles along the vertical where the maximum is detected at Mersch and Torre Accio cross-sections under different hydraulic conditions. 

As one can see, the maximum’s location was fairly stable and unaltered by maximum depth and flow rate variations, according to what is reported in the literature [45,50].

Furthermore, *u*_max_ was always detected below the free surface, even when the cross-section exhibited a large aspect ratio (see Table 1). The minimum observed *Y_d_* was 0.6. The situation was thus similar to that encountered during field measurements in large rivers such as the Mississippi, where the peak of velocity was observed at two-thirds of the water depth from the channel bottom [51]. 

**Figure 9:**
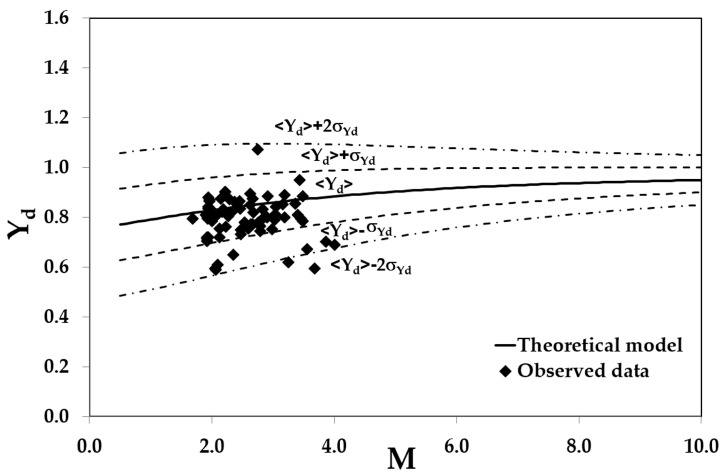
Predicted and measured dimensionless dip positions for different flow conditions at the investigated cross-sections.

In order to test the validity of the method proposed by the present study, a comparison between theoretical predictions based on Equations (24) and (25) and dimensionless dip positions measured for different depths and flow rates at all of the investigated cross sections is shown in Figure 9.

As one can see, most of the data are rather close to the theoretical ensemble mean; specifically, 87% of the data fall within the 95% confidence interval $<Y_{d}>\pm \sigma_{Y_{d}}$, which means that the proposed model well agrees with the experimental data over a wide range of channel aspect ratios. 

Model performance can also be compared with that by [26,27,29,33,34,39], whose explicit mathematical form is given in Table 3, based on four different error indicators:

$$\text{Average Percentage Relative Error}\;\left(\mathrm{APRE}\right)=\frac{1}{N}\sum_{i=1}^{N}\frac{\left|{\left(Y_{d,c}\right)}_{i}-{\left(Y_{d,m}\right)}_{i}\right|}{{\left(Y_{d,m}\right)}_{i}}\times 100\left(\%\right)$$

$$\text{Sum of squared relative error}\;\left(\mathrm{SSRE}\right)=\sum_{i=1}^{N}\frac{{\left[{\left(Y_{d,c}\right)}_{i}-{\left(Y_{d,m}\right)}_{i}\right]}^{2}}{{\left[{\left(Y_{d,c}\right)}_{i}\right]}^{2}}$$

$$\text{Sum of logarithmic deviation errorr}\;\left(\mathrm{SLDE}\right)=\sum_{i=1}^{N}{\left(log\left|{\left(Y_{d,c}\right)}_{i}\right|-log\left|{\left(Y_{d,m}\right)}_{i}\right|\right)}^{2}$$

$$\text{Root mean square error}\;\left(\mathrm{RMSE}\right)=\sqrt{\frac{1}{N}\sum_{i=1}^{N}{\left({\left(Y_{d,c}\right)}_{i}-{\left(Y_{d,m}\right)}_{i}\right)}^{2}}$$

where *N* indicates the total number of data, and *Y_d,c_* and *Y_d,m_* indicate the computed and observed velocity dip positions, respectively.

Due to its advantages of scale-independency and interpretability, the average percentage relative error (APRE) is one of the most used relative measures of model accuracy. However, its significant disadvantage is that it produces infinite or undefined values for zero or observed values that are close to zero. The sum of squared relative error (SSRE) works in a similar fashion, but the use of squared values makes it far more sensitive to larger than smaller relative errors. Conversely, being SLDE, the sum of the squares of the differences between the logarithm of predicted and observed values, it tends to emphasise low-magnitude errors. Finally, the root mean square error (RMSE) represents the standard deviation of the differences between predicted and observed values, takes into account the errors’s distribution, and it is very sensitive to outliers.

Overall, the use of all four error indicators enables providing a robust and complete evaluation of model performance and accuracy. Table 4 shows how the proposed entropy-based model is in any case characterised by the lowest error and greater accuracy, thus giving the best representation of the experimental measurements. 

## 5. Conclusions

Knowledge of magnitude and location of the maximum velocity in an open-channel cross-section can ease the data collection by reducing the needed number of samples, help understanding the related physical processes, and improve the design and the control of hydraulic engineering systems. 

Due to the occurrence of anisotropic turbulence and cross-sectional secondary currents, which tend to shift the maximum velocity from the free surface to the bed (with the shift that is relatively more pronounced in asymmetrical cross-sections of compound channels), its identification is still a complex task in hydraulics. 

A theoretical model leading to an efficient iterative evaluation procedure was here developed to simultaneously calculate maximum cross-sectional velocity and corresponding position, based on the conditional maximisation of the related information entropy. The model, which implies the knowledge of the channel flow rate, the geometric survey of the cross-section under consideration, and a very reduced number of (often expensive) velocity measurements, was tested against a large number of experimental data collected along different rivers located in Italy and in the Grand Duchy of Luxembourg.

The model proved to be able to completely identify the velocity peak under different flow and bed conditions. In fact, the theoretical dimensionless dip positions were in agreement with the experimental data over a wide range of channel aspect ratios (8.0 < *A_R_* < 92.5) at the 95% confidence level. The iterative procedure for the simultaneous evaluation of velocity dip position and corresponding peak velocity converged in all of the cases with less than four iterations and a relative error smaller than 0.001.

Finally, the accuracy of the model proposed by the present study was tested against other formulas available in the literature on the basis of a detailed error analysis, thus demonstrating that it was associated with the least relative error.

## Figures and Tables

**Table 1:** Ranges of mean depth and flow rate at the measurement sites.

Section	Hydraulic Radius *R_H_* (m)	Flow Rate *Q* (m^3^/s)	*A_R_* (*B*/*R_H_*)
Hunsdorf	0.3 ÷ 1.4	3.5 ÷ 31.8	9.4 ÷ 45.0
Lintgen	0.6 ÷ 1.8	9.6 ÷ 38.2	8.0 ÷ 21.1
Mersch	0.5 ÷ 1.6	2.3 ÷ 44.3	10.6 ÷ 17.7
Torre Accio	1.0 ÷ 3.5	1.1 ÷ 197.9	11.3 ÷ 26.6
P. La Marmora	0.3 ÷ 1.2	2.3 ÷ 34.9	16.9 ÷ 40.4
Pizzutello	0.1 ÷ 1.0	0.5 ÷ 25.0	11.5 ÷ 92.5

**Table 2:** Ranges of section-averaged velocity, maximum velocity, entropic parameter, and related entropy for the six investigated measurement sections.

Section	U	*u*_max_	*M*	*H*(*u*)
Hunsdorf	0.89 ÷ 1.63	1.26 ÷ 2.19	2.79 ÷ 3.43	−0.38 ÷ −0.27
Lintgen	1.06 ÷ 1.41	1.63 ÷ 2.15	1.94 ÷ 2.60	−0.24 ÷ −0.14
Mersch	0.49 ÷ 1.47	0.68 ÷ 2.21	1.69 ÷ 3.87	−0.46 ÷ −0.11
Torre Accio	0.09 ÷ 1.39	0.13 ÷ 1.98	1.92 ÷ 3.48	−0.39 ÷ −0.14
P. La Marmora	0.73 ÷ 1.46	1.12 ÷ 2.04	1.90 ÷ 3.41	−0.38 ÷ −0.14
Pizzutello	0.27 ÷ 1.83	0.41 ÷ 2.54	1.91 ÷ 4.00	−0.48 ÷ −0.14

**Table 3:** Tested formulas for velocity dip position.

Authors	Formula
Wang 2001	$Y_{d,c}=0.44+0.212\left(\frac{A_{r}}{2}\right)+0.05\sin\left(\frac{2\pi }{2.6}\frac{A_{r}}{2}\right)$
Yang 2004	$Y_{d,c}={\left[1+1.3\exp\left(-\frac{A_{r}}{2}\right)\right]}^{-1}$
Bonakdari 2008	$Y_{d}=\frac{42.4+A_{r}^{4.2}}{94.7+A_{r}^{4.2}}$
Guo 2013	$Y_{d,c}={\left[1+\exp\left\{-{\left(\frac{A_{r}}{\pi }\right)}^{1.5}\right\}\right]}^{-1}$
Pu 2013	$Y_{d,c}=\frac{40.1+A_{r}^{4.4}}{80.5+A_{r}^{4.4}}$
Kundu 2017	$Y_{d,c}=Y_{*}+\frac{1}{2L}\ln\left[1+\left(e^{L}-1\right)\left\{1-e^{-0.07A_{r}^{1.88}}\right\}\right]$
Proposed model	$<Y_{d,c}>=\frac{0.5M}{\exp\left(M\right)-1}\left[\exp\left(M\right)\left(\frac{1}{M}-\frac{1}{M^{2}}\right)+\frac{1}{M^{2}}\right]+0.5$

**Table 4:** Error estimates for the tested dip-position formulas. APRE: average percentage relative error; RMSE: root mean square error; SLDE: sum of logarithmic deviation error; SSRE: sum of squared relative error.

Formula	APRE (%)	SSRE	SLDE	RMSE
Wang 2001	228.03	32.69	20.11	2.27
Yang 2004	26.39	3.63	0.93	0.21
Bonakdari 2008	26.61	3.67	0.95	0.22
Guo 2013	26.58	3.67	0.95	0.22
Pu 2013	26.70	3.69	0.95	0.22
Kundu 2017	26.47 ÷ 26.66	3.66 ÷ 3.68	0.94 ÷ 0.95	0.22
Proposed model	7.29	0.95	0.23	0.09
